# CD40 Ligand–CD40 Interaction Is an Intermediary between Inflammation and Angiogenesis in Proliferative Diabetic Retinopathy

**DOI:** 10.3390/ijms242115582

**Published:** 2023-10-25

**Authors:** Ahmed M. Abu El-Asrar, Mohd I. Nawaz, Ajmal Ahmad, Luna Dillemans, Mairaj Siddiquei, Eef Allegaert, Priscilla W. Gikandi, Gert De Hertogh, Ghislain Opdenakker, Sofie Struyf

**Affiliations:** 1 Department of Ophthalmology, College of Medicine, King Saud University, Riyadh 11411, Saudi Arabia; mnawaz@ksu.edu.sa (M.I.N.); aajmal@ksu.edu.sa (A.A.); msiddiquei@ksu.edu.sa (M.S.); pgikandi.c@ksu.edu.sa (P.W.G.); ghislain.opdenakker@kuleuven.be (G.O.); 2 Dr. Nasser Al-Rashid Research Chair in Ophthalmology, College of Medicine, King Saud University, Riyadh 11411, Saudi Arabia; 3 Laboratory of Molecular Immunology, Department of Microbiology, Immunology and Transplantation, Rega Institute, University of Leuven, 3000 Leuven, Belgium; luna.dillemans@kuleuven.be (L.D.); sofie.struyf@kuleuven.be (S.S.); 4 Laboratory of Histochemistry and Cytochemistry, University of Leuven, 3000 Leuven, Belgium; eef.allegaert@kuleuven.be (E.A.); gert.dehertogh@uzleuven.be (G.D.H.); 5 University Hospitals UZ Gasthuisberg, 3000 Leuven, Belgium; 6 Laboratory of Immunobiology, Department of Microbiology, Immunology and Transplantation, Rega Institute, University of Leuven, 3000 Leuven, Belgium

**Keywords:** proliferative diabetic retinopathy, inflammation, angiogenesis, CD40 ligand, endothelial cells

## Abstract

We aimed to investigate the role of the CD40-CD40 ligand (CD40L) pathway in inflammation-mediated angiogenesis in proliferative diabetic retinopathy (PDR). We analyzed vitreous fluids and epiretinal fibrovascular membranes from PDR and nondiabetic patients, cultures of human retinal microvascular endothelial cells (HRMECs) and Müller glial cells and rat retinas with ELISA, immunohistochemistry, flow cytometry and Western blot analysis. Functional tests included measurement of blood–retinal barrier breakdown, in vitro angiogenesis and assessment of monocyte-HRMEC adherence. CD40L and CD40 levels were significantly increased in PDR vitreous samples. We demonstrated CD40L and CD40 expression in vascular endothelial cells, leukocytes and myofibroblasts in epiretinal membranes. Intravitreal administration of soluble (s)CD40L in normal rats significantly increased retinal vascular permeability and induced significant upregulation of phospho-ERK1/2, VEGF, intercellular adhesion molecule-1 (ICAM-1) and vascular cell adhesion molecule-1 (VCAM-1). sCD40L induced upregulation of VEGF, MMP-9, MCP-1 and HMGB1 in cultured Müller cells and phospo-ERK1/2, p65 subunit of NF-ĸB, VCAM-1 and VEGF in cultured HRMECS. TNF-α induced significant upregulation of CD40 in HRMECs and Müller cells and VEGF induced significant upregulation of CD40 in HRMECs. sCD40L induced proliferation and migration of HRMECs. We provide experimental evidence supporting the involvement of the CD40L-CD40 pathway and how it regulates inflammatory angiogenesis in PDR.

## 1. Introduction

Diabetic retinopathy (DR) is the most frequent microvascular complication of diabetes mellitus and is characterized by progressive retinal vasculopathy with endothelial cell dysfunction, breakdown of the blood–retinal barrier (BRB) and ischemia-induced retinal angiogenesis. Increased vascular permeability caused by the breakdown of the BRB results in diabetic macular edema, which represents the major cause of visual impairment in diabetic patients [1]. Angiogenesis and expansion of the extracellular matrix in association with the outgrowth of fibrovascular epiretinal membranes at the vitreoretinal interface are the hallmarks of proliferative diabetic retinopathy (PDR). Pathological fibrovascular tissue frequently leads to visual loss by two major mechanisms: bleeding into the vitreous and tractional retinal detachment. Chronic low-grade subclinical inflammation triggers many of the vascular lesions of DR. In rats, inflammation associated with increased retinal leukocyte stasis occurs within few days of developing streptozotocin-induced diabetes, a model of insulin-dependent diabetes mellitus. This leukostasis correlates with the increased retinal expression of intercellular adhesion-molecule-1 (ICAM-1) and the leukocyte integrin cluster of differentiation (CD)18. Diabetic endothelial cell damage, retinal vascular leakage and capillary nonperfusion are associated with retinal leukostasis. Blockade of the interaction between ICAM-1 and CD18 diminishes diabetic retinal leukostasis and vascular leakage [2].

In the ocular microenvironment of patients with PDR, several studies demonstrated the overexpression of various inflammatory and angiogenic factors, including secreted enzymes, cytokines, chemokines and membrane-anchored signaling proteins [3,4,5,6,7]. Epiretinal fibrovascular membranes from PDR patients contain new blood vessels, leukocytes and α-smooth muscle actin (α-SMA)-expressing myofibroblasts [3,4,5,6]. Epiretinal membrane leukocytes supply factors that foster angiogenesis [3,4,5,6] and link persistent inflammation with neovascularization in PDR initiation and progression. This causal relationship between persistent inflammation and angiogenesis is well established in several pathological conditions, such as cancer and vascular diseases [8,9]. In inflammation-driven angiogenesis, it has been shown that angiogenic cytokines or cell surface molecules expressed by infiltrating leukocytes can mediate the angiogenic response, a process called leukocyte-induced angiogenesis [9,10,11]. These findings suggest that leukocytes play important pathophysiologic roles in PDR initiation and progression.

Vascular endothelial growth factor (VEGF) is a key driver of retinal vascular leakage and angiogenesis [12]. VEGF exerts its effects by binding to its receptor VEGF-R2, which is expressed on vascular endothelial cells [13]. As inflammation is an initiating event in PDR, identification of biomarkers related to leukocyte activity and reflecting the extent of inflammation may provide insight into the molecular links between inflammation and angiogenesis and the cellular processes linked to PDR progression. In studies on inflammatory bowel and vascular diseases it is reported that the CD40-CD40 ligand (CD40L, also called CD154) signaling pathway crucially triggers inflammatory angiogenesis and mediates leukocyte-endothelial interactions [14,15,16,17].

CD40 is a type I transmembrane glycoprotein that belongs to the tumor necrosis factor (TNF)-receptor superfamily. It is expressed on the surface of antigen-presenting cells such as dendritic cells, B cells and monocytes/macrophages, as well as on other cell types, including vascular endothelial cells, epithelial cells and fibroblasts. CD40L, which is a member of the TNF superfamily, is a type II transmembrane protein expressed on activated T cells and platelets, and is also expressed on many other cell types, such as B cells, basophils, eosinophils, monocytes/macrophages, Kupffer cells, natural killer cells, mast cells, dendritic cells, fibroblasts, smooth muscle cells, epithelial cells and endothelial cells [15,16,17]. Interactions between CD40L and its counter-receptor CD40 have been found to have a fundamental role in inflammation and development of adaptive immunity against infections and cancer, predominantly in the effector phase of the immune response. This interaction promotes T cell activation, T cell-B cell interactions, B cell growth, differentiation and immunoglobulin class switching and secretion, T cell-dependent macrophage activation, as well as endothelial cell activation [15,16,17]. In addition to the cell-associated form, CD40L also exists in a soluble, biologically active form (sCD40L) which results from the shedding of the membrane-anchored CD40L. It was demonstrated that cleavage of the membrane-bound CD40L requires proteases of the family of a disintegrin and metalloproteinase (ADAM)10 and ADAM17 [18]. Similarly, a soluble form of CD40 (sCD40) is generated after processing of the membrane-anchored CD40 by proteolytic cleavage through ADAM17 [19]. Beyond its function on immune cells, the CD40L-CD40 signaling pathway mediates angiogenesis [14,20,21,22]. These data raise the interesting possibility that this pathway may be involved in critical events leading to initiation and progression of PDR. Therefore, the present study was designed to investigate the role of the CD40L-CD40 pathway in inflammation-driven angiogenesis in PDR.

## 2. Results

### 2.1. CD40 and CD40L Are Present in Vitreous Samples from Patients with PDR

**Figure 1:**
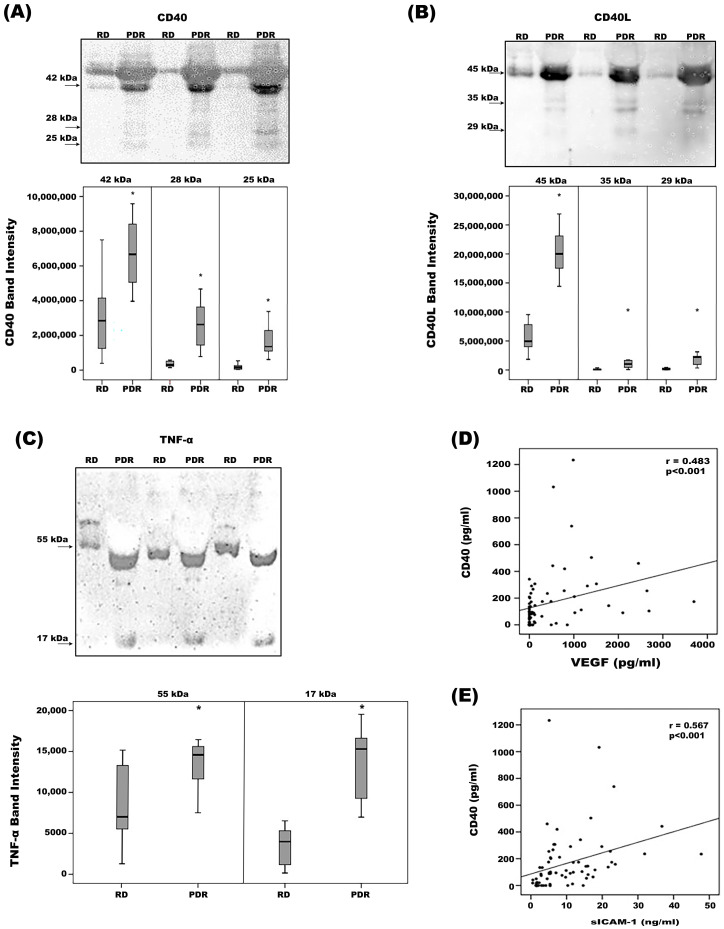
Determination of CD40 (**A**)**,** CD40L (**B**) and TNF-α (**C**) levels in vitreous fluid samples. Equal volumes (15 µL) of vitreous fluid samples from patients with proliferative diabetic retinopathy (PDR; *n* = 12) and from nondiabetic patients with rhegmatogenous retinal detachment (RD; *n* = 12) were subjected to gel electrophoresis and the presence of CD40, CD40L and TNF-α was detected by Western blot analysis. Representative sets of samples are shown. The intensity of the protein bands was determined in all samples. Band intensities were compared between RD and PDR groups. Results are expressed as median (interquartile range). (* *p* < 0.05; Mann–Whitney test). We also used ELISA to determine CD40, vascular endothelial growth factor (VEGF) and soluble intercellular adhesion molecule-1 (sICAM-1) levels in vitreous fluid samples from 38 PDR patients and 32 nondiabetic controls. Significant positive correlations between vitreous fluid ELISA levels of CD40 and the levels of VEGF (**D**) and sICAM-1 (**E**) (Spearman’s correlation coefficient).

With the use of Western blot analysis of equal volumes of vitreous fluid, we confirmed the presence of CD40 and CD40L in vitreous samples. CD40 immunoreactivities were observed as three protein bands at approximately 42 kDa, 28 kDa and 25 kDa (Figure 1A). The 28 kDa and 25 kDa forms might represent proteolytic fragments. CD40L immunoreactivities were expressed as three protein bands at approximately 45 kDa, 35 kDa and 29 kDa (Figure 1B). The 35 kDa and 29 kDa forms might represent soluble fragments. With the use of Western blot analysis, we also demonstrated that TNF-α was present in vitreous samples (Figure 1C). Scanning analysis of immunoreactivities demonstrated increased levels of all CD40 forms (Figure 1A), all CD40L forms (Figure 1B) and TNF-α (Figure 1C) in vitreous of PDR patients in comparison with controls.

We also used ELISA to compare CD40 levels in vitreous samples from 38 PDR patients with those of 32 nondiabetic controls. CD40 was detected in 22 of 32 (69%) vitreous samples from nondiabetic controls, and in all vitreous samples from PDR patients. CD40 levels in vitreous samples from PDR patients were significantly higher than the levels in nondiabetic controls (*p* < 0.001; Mann–Whitney test) (Table 1). To allow correlation analysis with ongoing inflammation and angiogenesis in the ocular microenvironment of patients with PDR, we also analyzed levels of VEGF (angiogenic biomarker) and sICAM-1 (proinflammatory biomarker). Both proteins were detected in all vitreous samples from PDR patients and levels were significantly enhanced compared to nondiabetic control samples (*p* < 0.001 and *p* = 0.001, respectively; Mann–Whitney test) (Table 1). Interestingly, significant positive correlations (Spearman’s correlation coefficient) were found between vitreous fluid levels of CD40 and levels of VEGF (r = 0.483; *p* < 0.001) (Figure 1D) and sICAM-1 (r = 0.567; *p* < 0.001) (Figure 1E).

### 2.2. Expression of CD40L and CD40 in Epiretinal Fibrovascular Membranes from Patients with PDR

**Figure 2:**
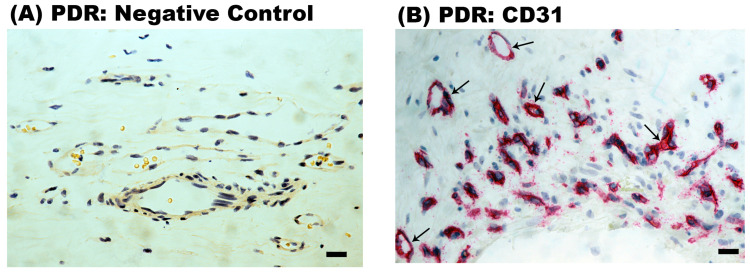

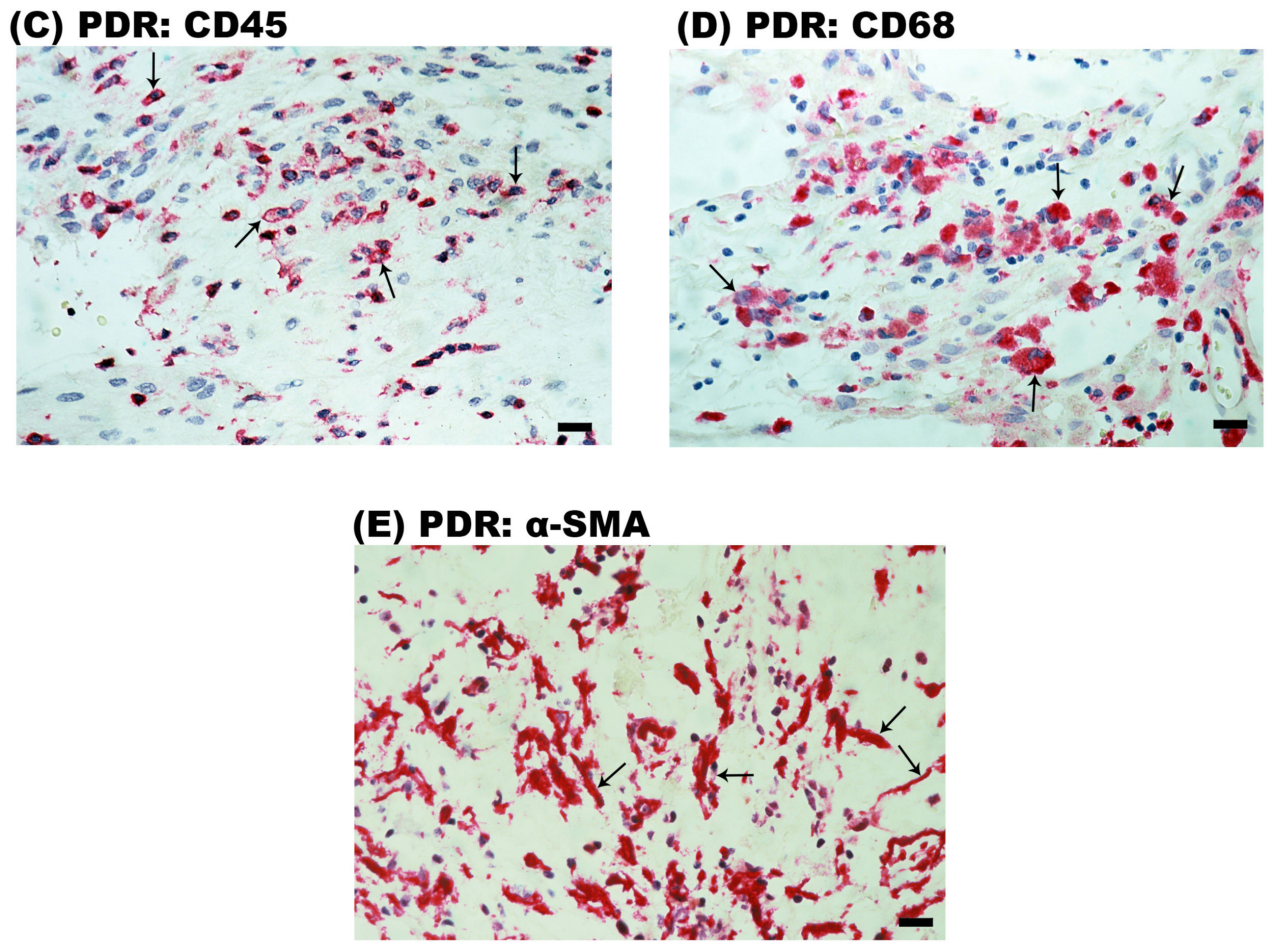
Immunohistochemical staining of proliferative diabetic retinopathy epiretinal fibrovascular membranes. (**A**) Negative control slide showing no labelling. Immunohistochemical staining for the endothelial cell marker CD31 showing pathologic new blood vessels (arrows) (**B**). Immunohistochemical staining for the leukocyte common antigen CD45 showing infiltrating leukocytes in the stroma (arrows) (**C**). Immunohistochemical staining for the monocyte/macrophage marker CD68 showing infiltrating monocytes/macrophages in the stroma (arrows) (**D**). Immunohistochemical staining for α-smooth muscle actin (α-SMA) showing immunoreactivity in spindle-shaped myofibroblasts (arrows) (**E**) (black scale bar, 10 µm).

**Figure 3:**
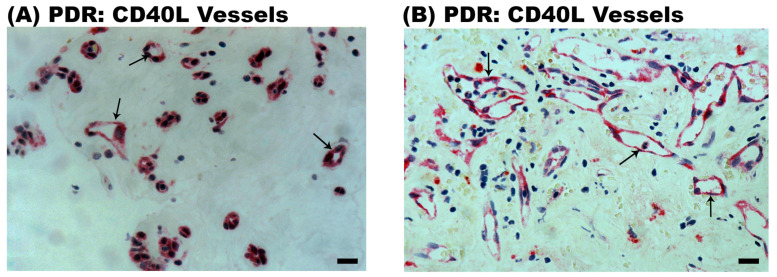

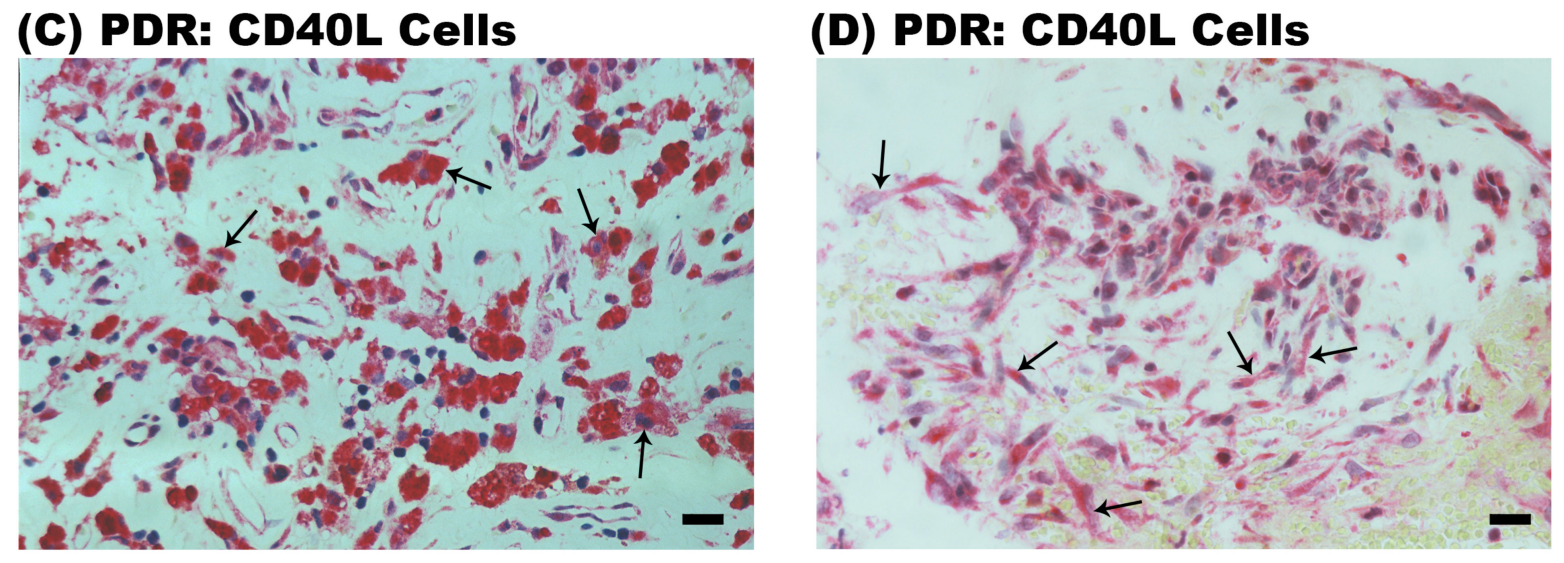
Immunohistochemical staining of proliferative diabetic retinopathy epiretinal fibrovascular membranes. Immunohistochemical staining for CD40L showing immunoreactivity in vascular endothelial cells (arrows) (**A**,**B**). Immunoreactivity for CD40L was also detected in stromal cells (arrows) (**C**) and in stromal spindle-shaped myofibroblasts (arrows) (**D**) (black scale bar, 10 µm).

**Figure 4:**
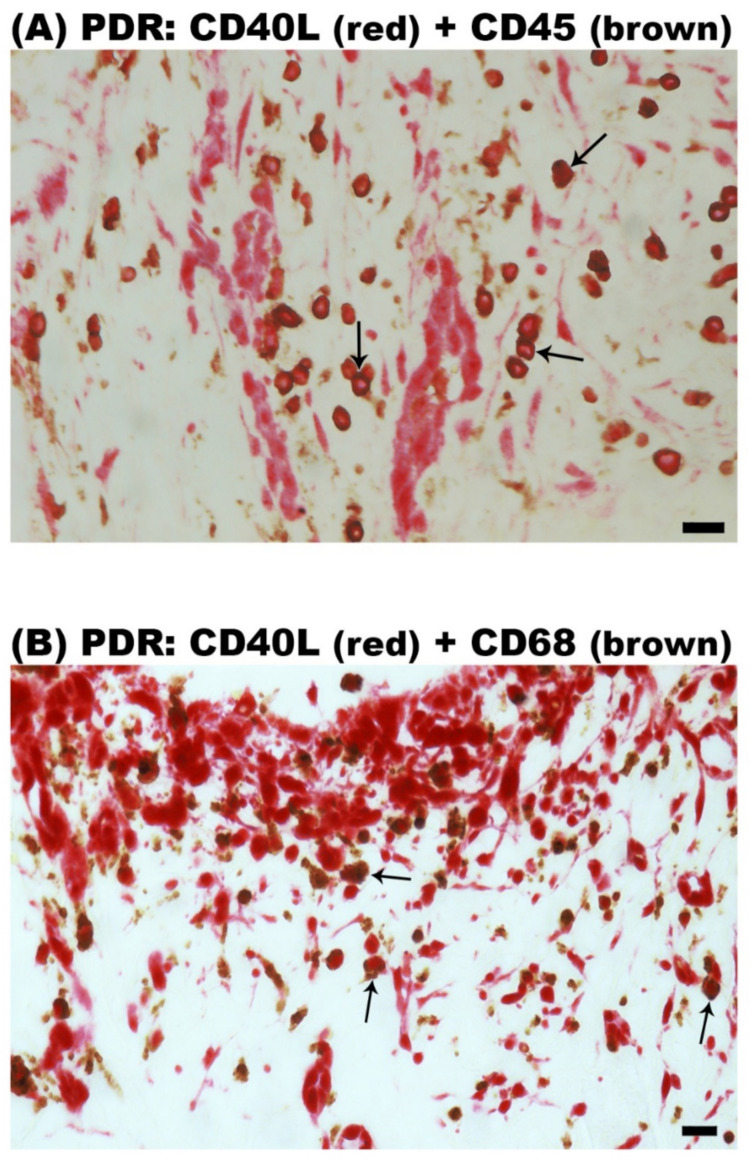
Immunohistochemical staining of proliferative diabetic retinopathy epiretinal fibrovascular membranes. Double immunohistochemical staining for CD40L (red) and CD45 (brown) (**A**) or CD68 (brown) (**B**) showing co-expression in stromal cells. No counterstain to visualize the cell nuclei was applied. Arrows indicate examples of double-stained cells.(black scale bar, 10 µm).

Epiretinal fibrovascular membranes from 14 patients with PDR were studied by immunohistochemical analysis to define producer cell types in vivo. No staining was observed in the negative control slides (Figure 2A). Neovessels, total leukocytes, monocyte/macrophages and myofibroblasts were detected by the corresponding markers CD31 (Figure 2B), CD45 (Figure 2C), CD68 (Figure 2D) and α-SMA (Figure 2E), respectively, as in our previous studies [3,4,5,6]. Immunoreactivity for CD40L was observed in all membranes in endothelial cells lining new blood vessels (Figure 3A,B) and stromal cells (Figure 3C,D). Stromal cells were leukocytes co-expressing CD45 (Figure 4A) and monocytes/macrophages co-expressing CD68 (Figure 4B). The stromal spindle-shaped cells expressing CD40L (Figure 3D) are most probably myofibroblasts, as their morphology is very similar to the α-SMA-expressing myofibroblasts shown in Figure 2E.

**Figure 5:**
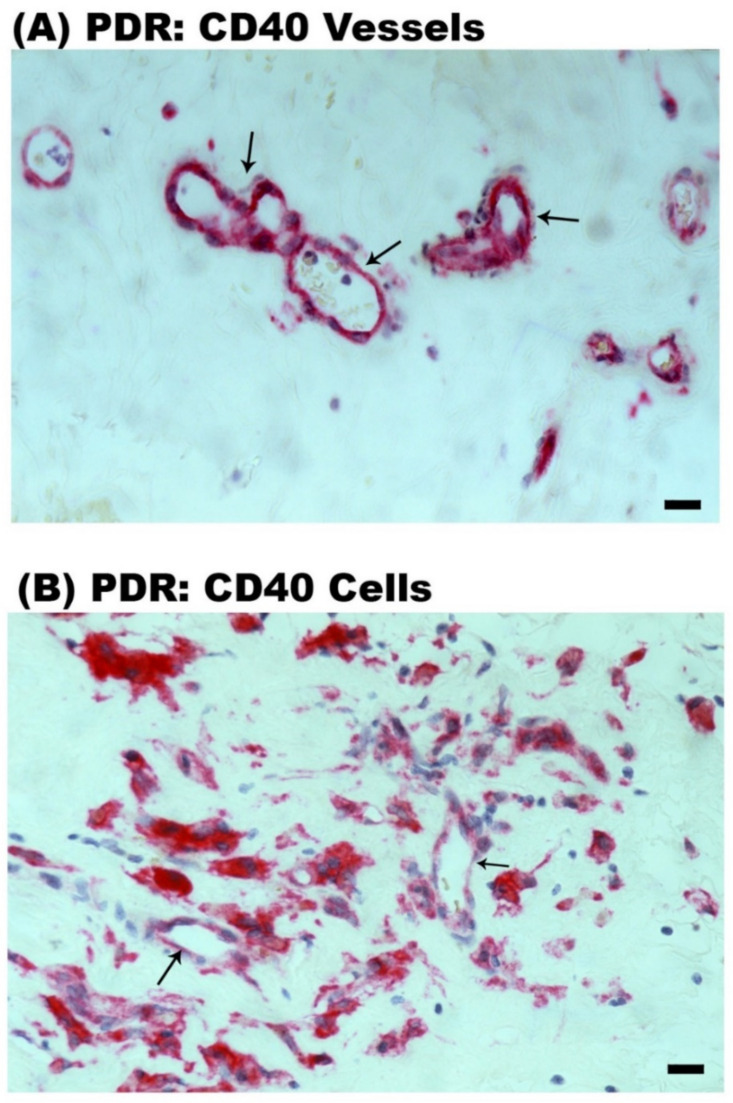
Immunohistochemical staining of proliferative diabetic retinopathy epiretinal fibrovascular membranes. Immunohistochemical staining for CD40 showing immunoreactivity in vascular endothelial cells (arrows) (**A**,**B**) and in stromal cells (**B**) (black scale bar, 10 µm).

**Figure 6:**
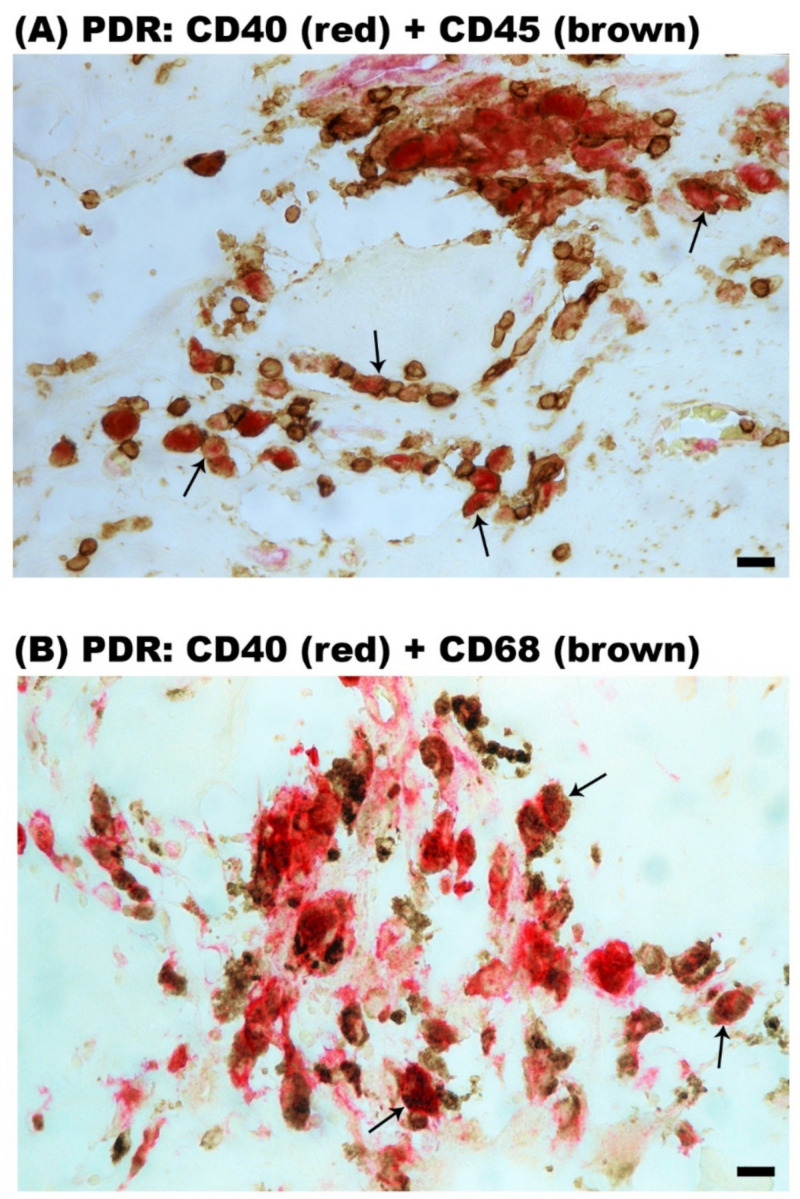
Immunohistochemical staining of proliferative diabetic retinopathy epiretinal fibrovascular membranes. Double immunohistochemical staining for CD40 (red) and CD 45 (brown) (**A**) or CD68 (brown) (**B**) demonstrating co-expression in stromal cells (arrows). No counterstain to visualize the cell nuclei was applied (black scale bar, 10 µm).

**Figure 7:**
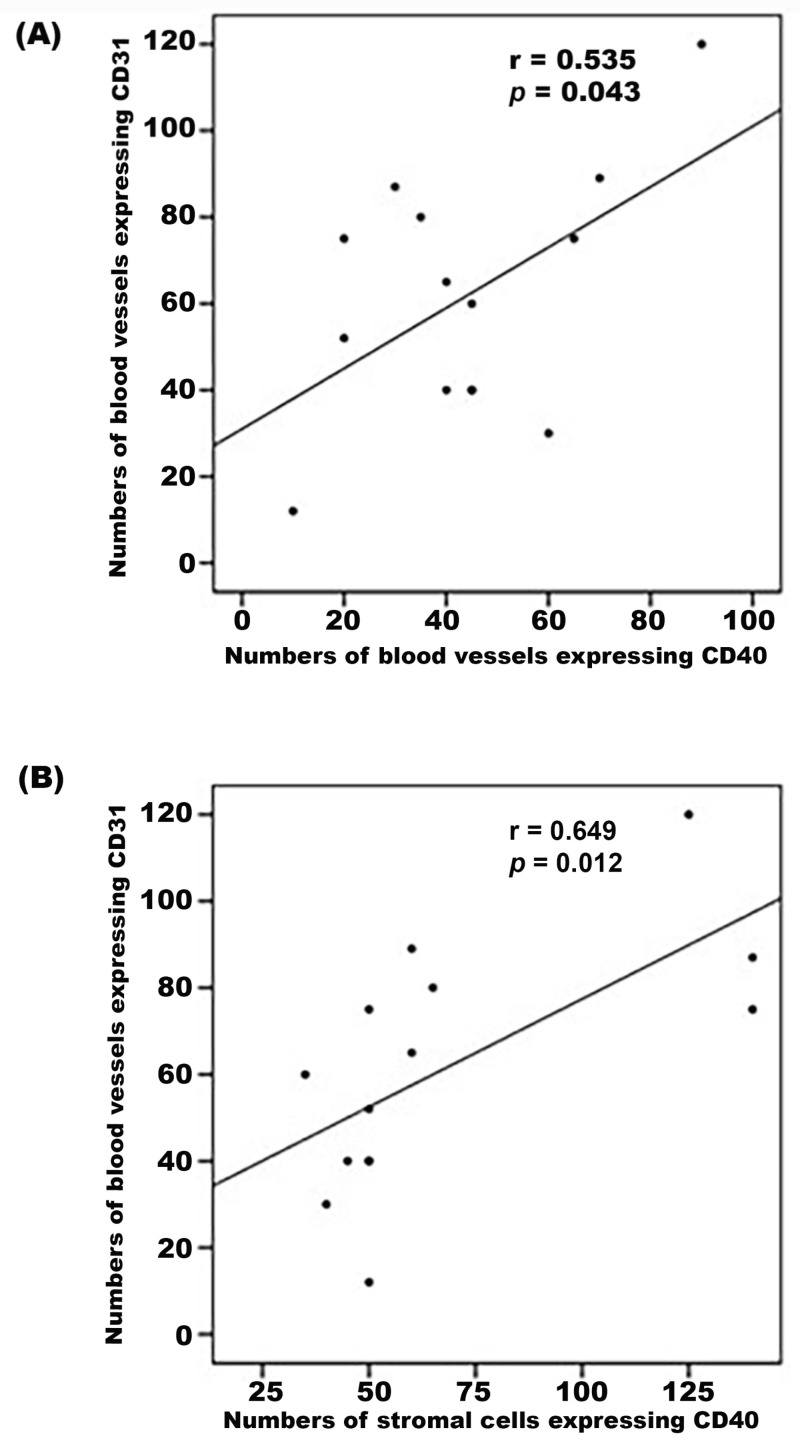
Epiretinal fibrovascular membranes from 13 patients with PDR were studied by immunohistochemical analysis. Significant positive correlations between the numbers of blood vessels expressing CD31 and the numbers of blood vessels expressing CD40 (**A**) and the numbers of stromal cells expressing CD40 (**B**) (Pearson’s correlation coefficient).

CD40 immunoreactivity was detected in vascular endothelial cells (Figure 5A,B) and stromal cells (Figure 5B). Similar to CD40L, CD40 was expressed by CD45-positive leukocytes (Figure 6A), and monocytes/macrophages co-expressing CD68 (Figure 6B). Significant positive correlations (Pearson’s correlation coefficient) were detected between the numbers of new blood vessels expressing CD31, reflecting the angiogenic activity of PDR epiretinal fibrovascular membranes, and the numbers of blood vessels (r = 0.535; *p* = 0.043) and stromal cells (r = 0.649; *p* = 0.012) expressing CD40 (Figure 7).

### 2.3. Effect of Intravitreal Administration of sCD40L in Normal Rats

**Figure 8:**
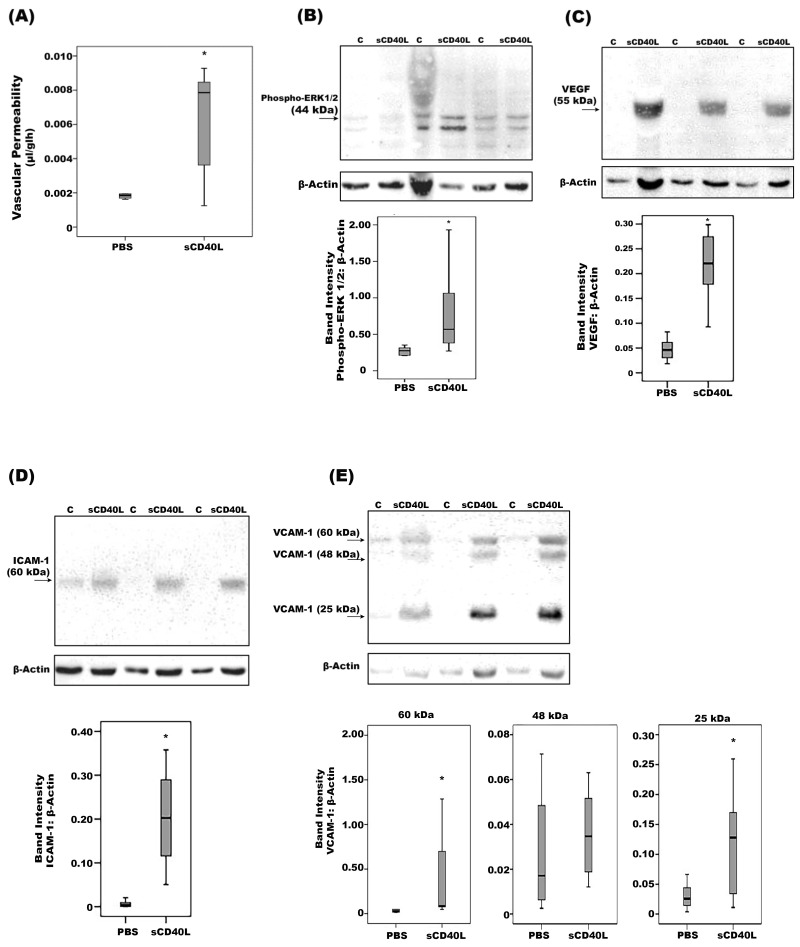
Soluble(s) CD40L induces blood-retinal barrier (BRB) breakdown. sCD40L was injected intravitreally at the dose of 5 ng in 5 µL in one eye and the same volume of phosphate-buffered saline (PBS) was injected in the contralateral eye of the normal rats. The BRB breakdown was quantified with the FITC-conjugated dextran technique. Results are expressed as median (interquartile range) of 8 rats (**A**). Western blot analysis of rat retinas. Intravitreal administration of sCD40L (5 ng in 5 µL) induced a significant upregulation of the expression of phospho-ERK1/2 (**B**), vascular endothelial growth factor (VEGF) (**C**), intercellular adhesion molecule-1 (ICAM-1) (**D**) and vascular cell adhesion molecule- 1 (VCAM-1) (**E**) compared to intravitreal administration of PBS (**C**). Results are expressed as median (interquartile range) of 10 rats. (* *p* < 0.05 compared to the values obtained from PBS-injected eyes; Mann–Whitney test).

To test whether the previous findings of the CD40/CD40L system are functionally relevant in vivo, we used a rat animal model. Intravitreal injection of sCD40L at a dose of 5 ng in 5 µL significantly increased retinal vascular permeability compared with PBS-injected eyes (Figure 8A). At doses of 1 ng in 5 µL or 2.5 ng in 5 µL, sCD40L did not significantly increase retinal vascular permeability compared with PBS-injected eyes. Western blot analysis of homogenized retinal tissue revealed that intravitreal administration of sCD40L induced significant upregulation of the protein levels of phospho-ERK1/2 (Figure 8B), VEGF (Figure 8C), ICAM-1 (Figure 8D) and VCAM-1 (Figure 8E), compared to the values obtained from the contralateral eye that was injected with vehicle (PBS).

### 2.4. Effect of Diabetic Retinopathy-Associated Mechanisms on the Expression of CD40 and CD40L in Human Retinal Müller Glial Cells

**Figure 9:**
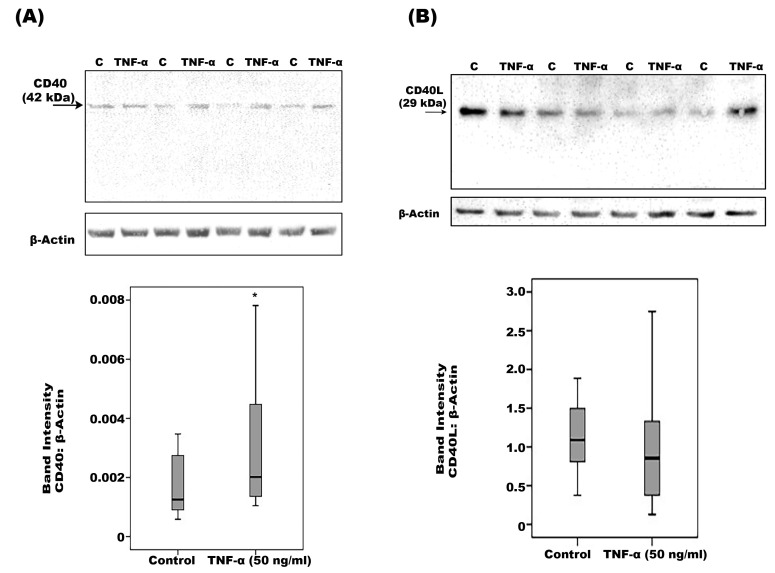
Müller cells were left untreated (C) or treated with tumor necrosis factor-α (TNF-α) for 24 h. Protein expression of CD40 (**A**) and CD40L (**B**) in the cell lysates was determined by Western blot analysis. Results are expressed as median (interquartile range) from three different experiments. (* *p* < 0.05 compared with the values obtained from untreated cells; Mann–Whitney test).

We next assessed the effect of the diabetic mimetic conditions high-glucose, the hypoxia-mimetic agent CoCl_2_, VEGF and TNF-α on the expression of CD40 and CD40L in human retinal Müller glial cells. With the use of Western blot analysis, we demonstrated that Müller cells constitutively express CD40 (Figure 9A) and CD40L (Figure 9B). Treatment with TNF-α induced significant upregulation of CD40 (Figure 9A), but not CD40L (Figure 9B). Treatment of Müller cells with high-glucose, CoCl_2_ or VEGF did not affect the expression of CD40 or CD40L. With ELISA analysis, we demonstrated that sCD40 remained below the detection limit in the culture medium and TNF-α did not increase its expression above this detection limit. This implies that Müller cells do not express the protease(s) that releases this antigen from the cell membrane.

### 2.5. Effect of CD40 Ligation on Human Retinal Müller Glial Cells

**Figure 10:**
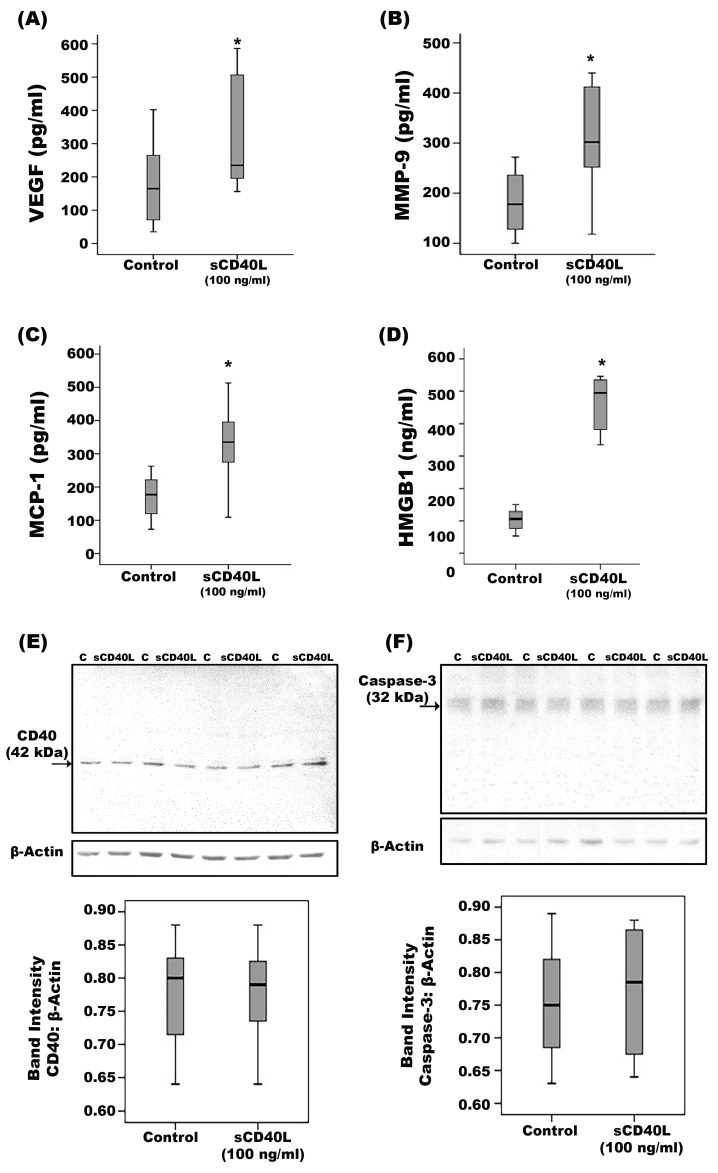

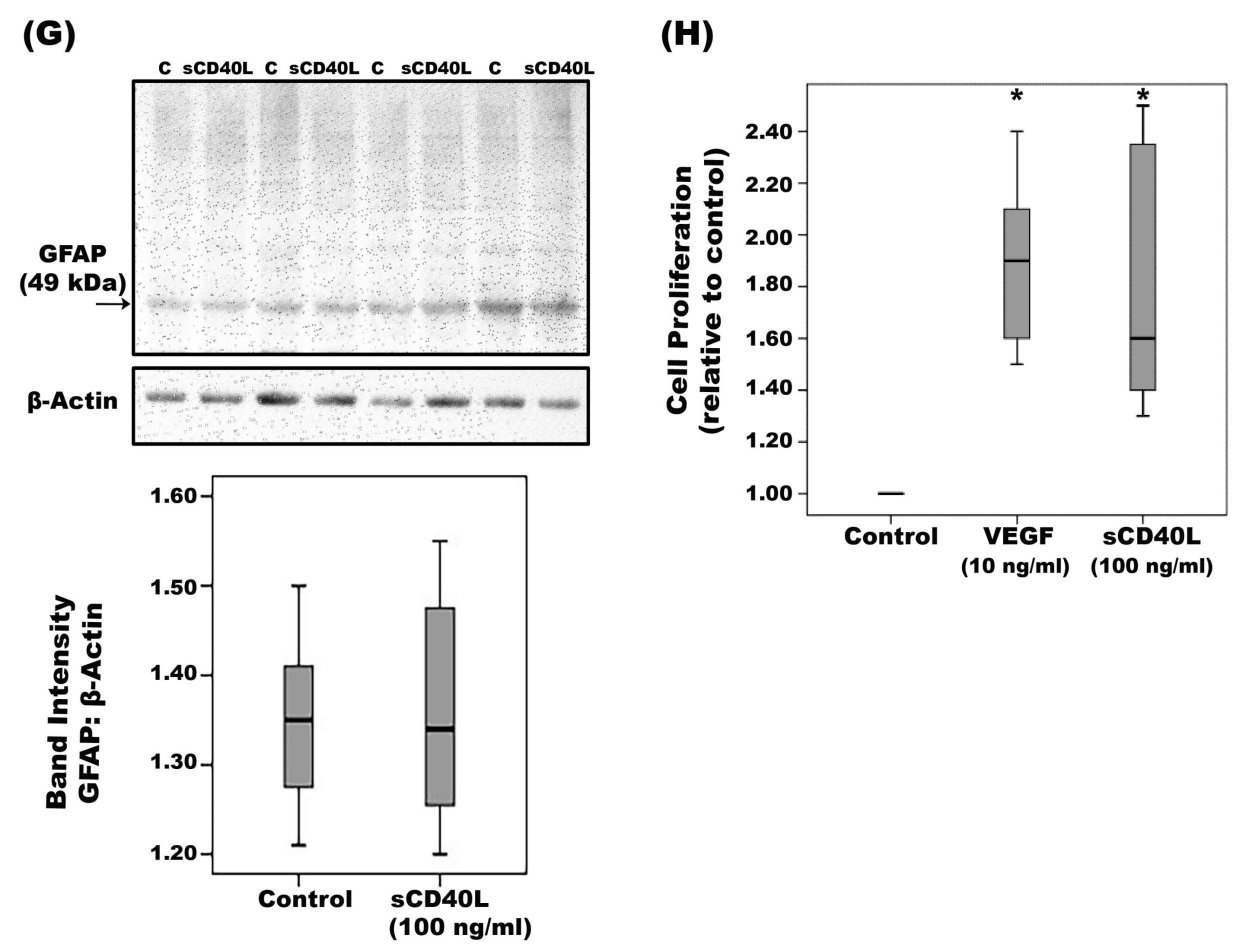
Müller cells were left untreated or treated with soluble CD40L for 24 h. Levels of vascular endothelial growth factor (VEGF) (**A**), matrix metalloproteinase-9 (MMP-9) (**B**), monocyte chemotactic protein (MCP)-1 (**C**) and high-mobility group box-1 (HMGB1) (**D**) were quantified in the culture media by ELISA. Protein expression of CD40 (**E**), caspase-3 (**F**) and glial fibrillary acidic protein (GFAP) (**G**) in the cell lysates was determined by Western blot analysis. sCD40L stimulated proliferation of Müller glial cells that was almost as potent as 10 ng/mL of VEGF compared to untreated cells (**H**). Results are expressed as median (interquartile range) from three different experiments. (* *p* < 0.05 compared with the values obtained from untreated cells; Mann–Whitney test).

In view of the infiltration of epiretinal fibrovascular membranes from patients with PDR with CD40L-expressing cells and increased levels of CD40L in vitreous samples from patients with PDR, we studied the effect of exogenous sCD40L on the expression of proangiogenic and inflammatory molecules in retinal Müller glial cells. Müller cells were cultured in the absence or presence of sCD40L. ELISA analysis revealed that exogenous sCD40L significantly increased the levels of the proangiogenic factors VEGF (Figure 10A) and MMP-9 (Figure 10B), the inflammatory chemokine MCP-1 (Figure 10C) and the potent proinflammatory alarmin HMGB1 (Figure 10D) in the culture medium as compared to untreated control. Treatment of Müller cells with sCD40L did not affect the expression of CD40 (Figure 10E), the apoptosis executer enzyme caspase-3 (Figure 10F) or the Müller cell activation marker glial fibrillary acidic protein (GFAP) (Figure 10G). Neither did treatment of Müller cells with sCD40L affect the signal transduction effectors phospo-ERK1/2 and NF-ĸB. In proliferation assays, sCD40L (100 ng/mL) induced proliferation of Müller glial cells. sCD40L was almost as potent as 10 ng/mL of VEGF (Figure 10H).

### 2.6. Effect of Diabetic Retinopathy-Associated Mechanisms on the Expression of CD40 and CD40L in Human Retinal Microvascular Endothelial Cells

**Figure 11:**
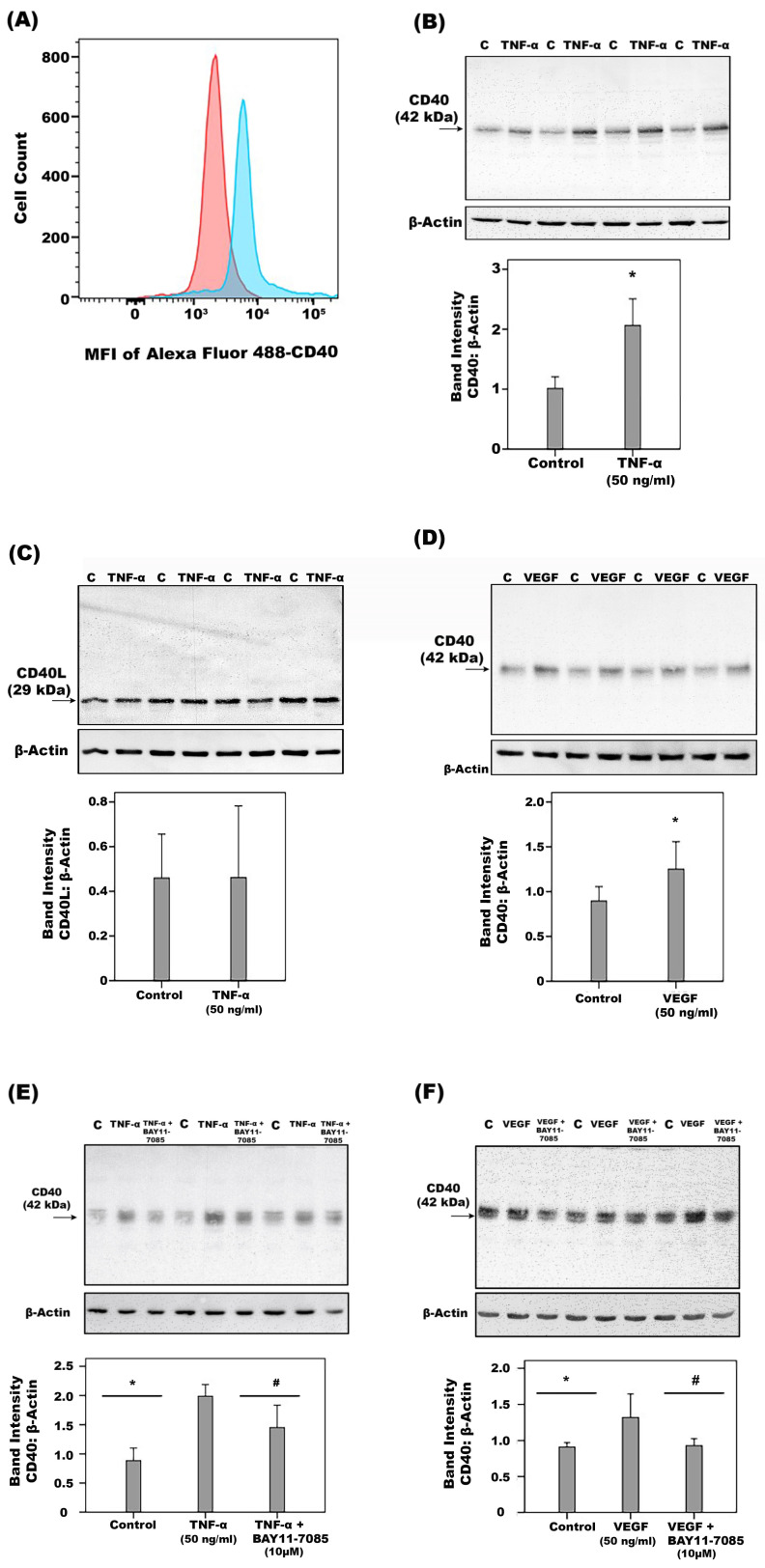
CD40 expression on human retinal microvascular endothelial cells (HRMECs) was evaluated through flow cytometry. Representative histogram of 4 independent experiments showing mean fluorescence intensity (MFI) of CD40 expression on HRMECs. CD40 expression on HRMECs stained with the primary mouse anti-human CD40 antibody and secondary goat anti-mouse AlexaFluor488 (blue) compared to cells incubated with the secondary antibody only (red) (**A**). HRMECs were left untreated (Control “C”) or treated with tumor necrosis factor-α (TNF-α), vascular endothelial growth factor (VEGF), TNF-α plus a 1 h-pre-incubation with BAY11-7085 or VEGF plus a 1 h-pre-incubation with BAY11-7085 for 24 h. Protein expression levels of CD40 (**B**,**D**–**F**) and CD40L (**C**) in cell lysate were determined by Western blot analysis. Results are expressed as mean ± standard deviation from three different experiments. One-way ANOVA and independent *t*-test were used for comparisons between three groups and two groups, respectively. (* *p* < 0.05 compared with the values obtained from untreated cells. # *p* < 0.05 compared with TNF-α-or VEGF-treated cells).

**Figure 12:**
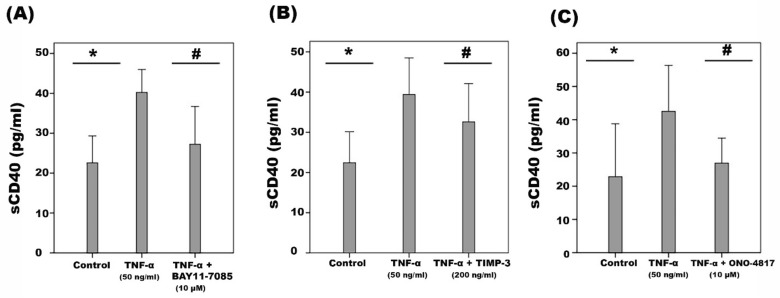
Human retinal microvascular endothelial cells were left untreated or were stimulated with tumor necrosis factor-α (TNF-α) for 24 h or TNF-α plus pre-incubation with BAY11-7085 (**A**), tissue inhibitor of metalloproteinase-3 (TIMP- 3) (**B**) or ONO-4817 (**C**). Levels of soluble (s) CD40 were quantified in the culture media by ELISA. Results are expressed as mean ± standard deviation from three different experiments. One-way ANOVA and independent *t*-test were used for comparisons between three groups and two groups, respectively. (* *p* < 0.05 compared with the values obtained from untreated cells. # *p* < 0.05 compared with TNF-α-treated cells).

Flow cytometry (Figure 11A) and Western blot analysis of cell lysates confirmed that HRMECs constitutively express CD40 (Figure 11B) and CD40L (Figure 11C). Furthermore, TNF-α (Figure 11B) and VEGF (Figure 11D) significantly increased the expression of CD40, but not CD40L (Figure 11C). Interestingly, co-treatment of HRMECs with TNF-α (Figure 11E) or VEGF (Figure 11F) plus the NF-ĸB inhibitor BAY11-7085 significantly attenuated TNF-α- or VEGF-induced upregulation of CD40, indicating that upregulation of CD40 in response to those immune modulators is dependent on the NF-ĸB signaling pathway. High-glucose or CoCl_2_ did not affect the expression of CD40 and CD40L. Regulation of CD40 expression in HRMECs by TNF-α and NF-κB was confirmed by ELISA detection of sCD40 in the cell culture supernatants (Figure 12A). We also applied protease inhibitors to identify the type of protease shedding CD40 from the cell membrane. Co-treatment with TNF-α plus TIMP-3 (Figure 12B) or ONO-4817 (Figure 12C) significantly attenuated TNF-α-induced upregulation of sCD40 in HRMECs, indicating that TNF-α-promoted CD40 shedding is dependent on MMPs. In contrast, dec-CMK (the proprotein convertase furin inhibitor) did not affect TNF-α-induced release of sCD40.

Treatment of HRMECs with TNF-α significantly upregulated the adherence of monocytes to HRMECs, whereas sCD40L did not affect their adherence to HRMECs. Moreover, the CD40-CD40L inhibitor DRI-C21045 did not affect TNF-α-induced increased adherence of monocytes to HRMECs.

### 2.7. Effect of Ligation of CD40 on Human Retinal Microvascular Endothelial Cells

**Figure 13:**
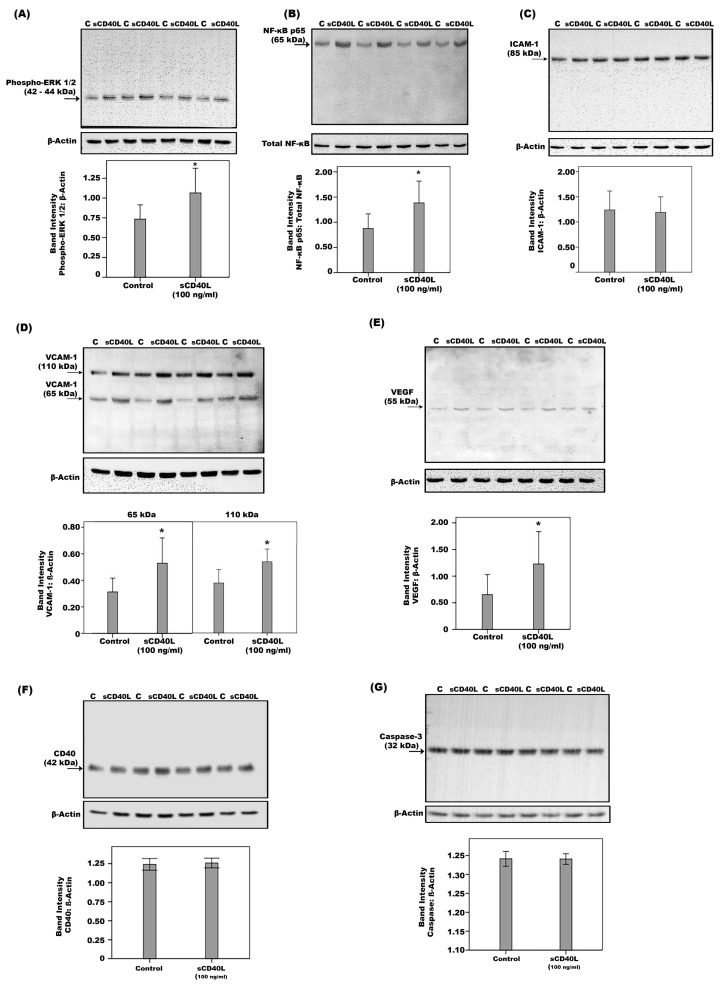
Human retinal microvascular endothelial cells were left untreated (C) or were stimulated with sCD40L for 24 h. Protein expression of phospho-ERK1/2 (**A**), the p65 subunit of NF-ĸß (**B**), intercellular adhesion molecule-1 (ICAM-1) (**C**), vascular cell adhesion molecule-1 (VCAM-1) (**D**), vascular endothelial growth factor (VEGF) (**E**), CD40 (**F**) and caspase-3 (**G**) in the cell lysates was determined by Western blot analysis. Results are expressed as mean ± standard deviation from three experiments (* *p* < 0.05; independent *t*-test).

Western blot analysis demonstrated that treatment of HRMECs with sCD40L induced significant upregulation of phospho-ERK1/2 (Figure 13A), the p65 subunit of NF-ĸB (Figure 13B), VCAM-1 (Figure 13D), and VEGF (Figure 13E). Exposure of HRMECs to sCD40L did not affect the expression of ICAM-1 (Figure 13C), CD40 (Figure 13F) or caspase-3 (Figure 13G).

**Figure 14:**
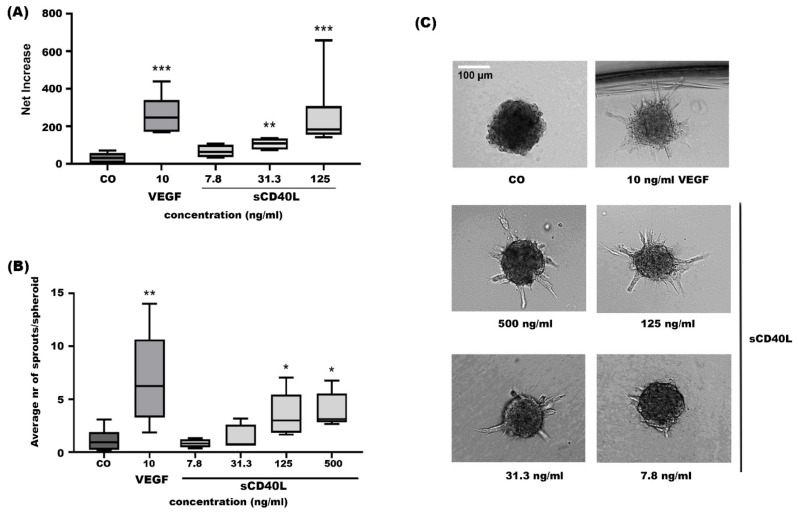
sCD40L stimulates proliferation and spheroid sprouting of HRMECs in vitro. (**A**) Net increase in the proliferation of HRMECs after stimulation with 10 ng/mL VEGF165 or 7.8 ng/mL, 31.3 ng/mL, or 125 ng/mL sCD40L compared to untreated cells (incubated with control medium EBM-2 + 1% FCS; CO). The data are displayed as a median (±IQR) of four to seven independent experiments. A Mann–Whitney test was performed to compare stimulated conditions to untreated cells (** *p* < 0.01, *** *p* < 0.001) and VEGF165 with sCD40L at 125 ng/mL (ns; not significant). (**B**) Average number of sprouts per spheroid. (**C**) Representative images of spheroids that were left untreated (incubated with control medium EBM-2 + 3% FCS; CO), incubated with 10 ng/mL VEGF165 or 7.8 ng/mL, 31.3 ng/mL, 125 ng/mL, or 500 ng/mL sCD40L are displayed. White scale bar = 100 µm. The data are displayed as a median (± IQR) of four to six independent experiments. A Mann–Whitney test was performed to compare stimulated conditions to untreated cells (* *p* < 0.05, ** *p* < 0.01) and VEGF165 with sCD40L at 500 ng/mL (ns; not significant).

In proliferation assays, sCD40L induced proliferation of HRMECs in a dose-dependent manner, with 31 ng/mL being the minimal effective concentration (Figure 14A). At the highest dose tested, sCD40L was almost as potent as 10 ng/mL of VEGF. During angiogenesis, endothelial cells do not only proliferate, they also migrate to reorganize into small new vessels. Therefore, we also tested sCD40L in sprouting assays with endothelial spheroids (Figure 14B,C). sCD40L was slightly less potent in this assay, as at least 125 ng/mL was required to induce a significant effect. The number of sprouts/spheroids was also lower than in response to 10 ng/mL of VEGF (Figure 14B).

## 3. Discussion

**Figure 15:**
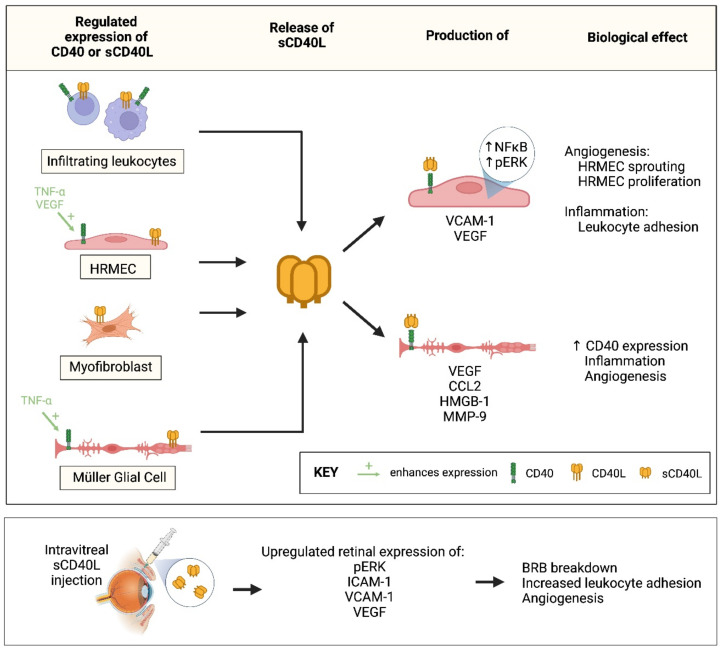
Summary of the in vitro and in vivo experiments that documented the relevance of the CD40/CD40L pathway in proliferative diabetic retinopathy. TNF-α = tumor necrosis factor-α; VEGF = vascular endothelial growth factor; HRMEC = human retinal microvascular endothelial cell; ERK = extracellular-signal regulated kinase; ICAM-1 = intercellular adhesion molecule-1; VCAM-1 = vascular cell adhesion molecule-1; NF-ĸB = nuclear factor-Kappa B; MCP-1 = monocyte chemotactic protein-1; HMGB1 = high mobility group box-1; MMP-9 = matrix metalloproteinase-9; BRB = blood–retinal barrier; ↑ = enhanced.

Inflammation and angiogenesis are processes involved in the pathogenesis of PDR, and the interplay between these events is under intense investigation [3,4,5,6,7]. The expression of CD40 and CD40L has been previously reported to be prominent in pathologic processes known to be associated with chronic inflammation and angiogenesis, such as cancer and inflammatory bowel and vascular diseases. Furthermore, blockade of CD40L-CD40 interactions has been found to inhibit these diseases [15,16,17]. The current study was designed to investigate the role of the CD40L-CD40 pathway in inflammation-driven angiogenesis in PDR. The schematic in Figure 15 illustrates the various levels of ex vivo, in vivo and in vitro experiments with which we documented the relevance of the CD40/CD40L system for PDR. We demonstrated that CD40L and CD40 levels were significantly upregulated in vitreous fluid samples and the membrane forms of CD40L and CD40 were expressed in epiretinal fibrovascular membranes from patients with PDR. Immunohistochemical analysis demonstrated CD40L protein expression by myofibroblasts and co-expression of CD40L and CD40 proteins in leukocytes and endothelial cells lining pathologic new blood vessels in the epiretinal membranes. It has been reported that sCD40L and sCD40 are produced locally as a result of proteolytic cleavage of the membrane-bound forms by the metalloproteinases ADAM10 and ADAM17 [18,19]. In a previous study, we demonstrated increased levels of ADAM10 and ADAM17 in the ocular microenvironment of patients with PDR [4], indicating that the detected membrane-bound CD40/CD40L might be shed from the endothelial cells, leukocytes and myofibroblasts. In addition, angiogenic activity (CD31-positive vessels) and CD40 expression in PDR fibrovascular epiretinal membranes showed a significant correlation.

Additional positive correlations were demonstrated between vitreous CD40 levels and angiogenic (VEGF) and inflammatory (sICAM-1) biomarkers, suggesting that CD40 may serve as a novel biomarker of inflammation and angiogenesis in the ocular microenvironment of patients with PDR. Previous studies demonstrated a critical role for CD40 in the pathogenesis of diabetes-induced retinal inflammation. Upregulation of CD40 occurred in the retina of streptozotocin-induced diabetic mice. Deletion of CD40 in diabetic mice decreased inflammatory responses linked to the pathogenesis of diabetic retinopathy, such as retinal leukostasis, capillary degeneration, ICAM-1 upregulation and protein nitration [23,24]. In the current study, we demonstrated the functionality of the CD40/CD40L system in vivo, as we observed several pathology-related responses upon intravitreal injection of sCD40L in normal rats: upregulation of phospho-ERK1/2 and the leukocyte adhesion molecules ICAM-1 and VCAM-1 and breakdown of the BRB through upregulation of VEGF, a key player in BRB breakdown [12] and pathological retinal neovascularization and development of PDR [25].

Upregulation of CD40L and CD40 in the ocular microenvironment of patients with PDR strongly suggests that the CD40L-CD40 interaction is involved in the immunopathogenesis of PDR. Therefore, we investigated the effect of CD40 ligation using sCD40L stimulation of retinal cells. Retinal Müller glial cells and HRMECs are major cell types which actively participate in diabetes-induced inflammatory reactions in the retina [3,4,5,6]. For instance, Müller cells are a major source of VEGF [25]. We demonstrated that Müller cells and HRMECs constitutively express CD40L and CD40. In response to sCD40L, treated Müller cells secreted the proinflammatory alarmin HMGB1, the inflammatory chemokine MCP-1/CCL2 and the proangiogenic factors VEGF and MMP-9 in the culture supernatants. Additionally, sCD40L stimulation promoted phosphorylation of ERK1/2, activation of NF-ĸB and expression of VCAM-1 and VEGF in HRMECs. These observations imply that in the intraocular microenvironment of patients with PDR, ligation of CD40 with CD40L is a pathophysiological contributor to both inflammation and angiogenesis. Similarly, a previous study demonstrated that CD40 ligation induced ICAM-1 and MCP-1/CCL2 upregulation in retinal endothelial cells and Müller cells. Additionally, CD40 ligation upregulated VEGF production by Müller cells [26]. Our findings are thus consistent with previous reports documenting the role of sCD40L/CD40 interaction in the activation of ERK1/2 and NF-ĸB [27,28,29,30,31,32] and upregulation of VEGF [14,20,21,33,34,35,36], MMP-9 [15,17,37], ICAM-1 [28,29,30] and VCAM-1 [32] in different types of cells.

The adhesion of circulating leukocytes to retinal endothelial cells contributes to the inflammatory aspects of the progression of DR [2]. In a previous report, sCD40L treatment induced the expression of VCAM-1 in cultured human aortic endothelial cells and increased adherence of human monocytic THP-1 cells to these cells [32]. However, we did not observe enhanced adhesion of monocytic THP-1 cells to HRMECs upon exposure to sCD40L. It has been demonstrated that endothelial cells are heterogeneous in their response to sCD40L. Indeed, human aortic endothelial cells and human umbilical vein endothelial cells upregulated membrane-bound and soluble CX3CL1 and TNF-α in response to CD40 ligation. In contrast, human retinal endothelial cells did not upregulate CX3CL1, nor secrete TNF-α upon CD40 ligation [38]. In addition, depending on the cell type, interaction between CD40L and its counter-receptor, CD40, results in the activation of different signaling pathways [27,28,29,30,31,32,33].

Several reports demonstrated enhanced circulating levels of sCD40L in patients with diabetes mellitus [39,40,41,42]. sCD40L levels showed a positive correlation with hemoglobinA1c and plasma fasting glucose [40,41]. In addition, improved metabolic control was associated with a reduction in circulating sCD40L levels [39,41,42]. In the present study, we investigated the effect of various diabetes-associated mechanisms such as high-glucose, the hypoxia mimetic agent CoCl_2_, VEGF and the proinflammatory cytokine TNF-α on the expression of membrane-bound CD40 and sCD40. Among the diabetes-associated mechanisms studied, only TNF-α induced significant upregulation of membrane-bound CD40 expression on Müller cells and HRMECs. In addition, VEGF induced significant upregulation of membrane-bound CD40 expression on HRMECs. For both inducers, activation of the NF-ĸB signaling pathway was involved, as the NF-ĸB inhibitor BAY11-7085 attenuated TNF-α- or VEGF-induced upregulation of CD40. Constitutive and low levels of sCD40 were detected in the culture supernatants of HRMECs, but not Müller cells. Treatment of HRMECs, but not Müller cells, with TNF-α induced significant upregulation of sCD40 in the culture medium. Addition of the potent broad-spectrum MMP inhibitor ONO-4817, TIMP-3 and the NF-ĸB inhibitor BAY11-7085 attenuated TNF-α-induced upregulation of sCD40 in the culture supernatants of HRMECs. Our findings are in line with those of a previous report documenting the role of the metalloproteinase ADAM17 in proteolytic cleavage of the membrane-bound CD40 [19]. Our findings are also consistent with previous studies demonstrating that TNF-α can promote CD40 expression through activation of the NF-ĸB signaling pathway [17,28,31,43].

In addition to its function on immune cells, CD40 signaling triggered by CD40L has been found to mediate angiogenesis and CD40 expression is upregulated in angiogenic processes [15]. We demonstrated that ligation of CD40 on HRMECs and Müller cells with sCD40L promotes the expression of the proangiogenic factors MMP-9 and VEGF, key angiogenic factors in PDR [6,12]. In addition, we demonstrated that sCD40L stimulated proliferation and migration of HRMECs, key steps in the angiogenesis cascade, thereby confirming previous reports documenting sCD40L-mediated resistance to apoptosis and in vitro migration, proliferation and vessel-like structure formation by human umbilical vein endothelial cells and human intestinal microvascular endothelial cells in a VEGF-dependent manner [14,21,22]. Furthermore, subcutaneous injection of sCD40L in animal models enhanced VEGF expression and induced marked angiogenesis in vivo [20,21]. These findings define that CD40L-CD40 interactions promote VEGF expression and angiogenesis.

## 4. Materials and Methods

### 4.1. Patient Samples

During therapeutic pars plana vitrectomy for the treatment of tractional retinal detachment, and/or nonclearing vitreous hemorrhage, undiluted vitreous fluid samples were obtained from 38 PDR patients. These samples were processed as described previously [3,4,5,6]. The clinical control group of samples originated from 32 patients who underwent vitrectomy for the treatment of rhegmatogenous retinal detachment with no proliferative vitreoretinopathy. These control subjects were clinically checked to be free from diabetes or other systemic diseases. Epiretinal fibrovascular membranes were obtained from 14 patients with PDR during pars plana vitrectomy for the repair of tractional retinal detachment and processed as described [3,4,5,6,7]. Membranes were fixed for 2h in 10% formalin solution and embedded in paraffin.

### 4.2. Immunohistochemical Staining of Human Epiretinal Membranes and Quantifications

CD31, CD45, CD68 and α-SMA were detected as described [3,4,5,6]. For CD40L and CD40 detection, antigen retrievals were performed by boiling the sections in Tris/EDTA buffer [pH 9] [BOND Epitope Retrieval Solution 2; Leica, Wetzlar, Germany] for 20 min. Subsequently, the sections were incubated for 60 min with rabbit polyclonal anti-CD40L antibody (1:50; ab65854, Abcam, Cambridge, UK) and mouse monoclonal anti-CD40 antibody (1:1000, AMAb 90905, Atlas Antibodies, Bromma, Sweden). The sections were then incubated for 20 min with an IgG against the primary antibody and conjugated with alkaline phosphatase. The reaction product was visualized by incubation for 15 min with the Fast Red chromogen, resulting in bright red immunoreactive sites. The slides were then faintly counterstained with Mayer’s hematoxylin [BOND Polymer Refine Red Detection Kit; Leica].

To identify the phenotype of cells expressing CD40L and CD40, sequential double immunohistochemistry was performed. The sections were incubated with the first primary antibodies (anti-CD45 or anti-CD68) and subsequently treated with a peroxidase-conjugated secondary antibody to define the leukocytes. The resulting immune complexes were visualized by enzymatic reaction of the 3, 3′-diaminobenzidine tetrahydrochloride substrate, yielding brown precipitates. Incubation with anti-CD40L or anti-CD40 as primary antibodies was followed by treatment with an alkaline phosphatase-conjugated secondary antibody and Fast Red reactions, as indicated above. No counterstain was applied. In negative control experiments, the incubation step with primary antibodies was omitted from the staining protocol. Instead, the ready-to-use DAKO Real antibody Diluent (Agilent Technologies Product Code 52022, Santa Clara, CA, USA) was applied.

The level of vascularization in epiretinal membranes was determined by immunodetection of the vascular endothelium marker CD31. Immunoreactive blood vessels and cells were counted in five representative fields, with the use of an eyepiece with a calibrated grid in combination with the 40× objective. These representative fields were selected based on the presence of immunoreactive blood vessels and cells. With the used magnification and calibration, immunoreactive blood vessels and cells present in an area of 0.33 mm x 0.22 mm were counted.

### 4.3. Intravitreal Injection of sCD40L in Rats

Adult male Wistar rats 8–9 weeks of age (200–220 g) were kept under deep anesthesia, and sterilized solution of recombinant human sCD40L (1 ng in 5 µL, 2.5 ng in 5 µL or 5 ng in 5 µL; Cat No. 6420-CL, R&D Systems, Minneapolis, MN, USA) was injected into the vitreous of the right eye as previously described [3]. For the control, the left eye received 5 μL of sterile phosphate-buffered saline (PBS). The animals were sacrificed 5 days after intravitreal administration, and the retinas were carefully dissected, snap frozen in liquid nitrogen, and stored at −80 °C until analyzed.

### 4.4. Measurement of Blood-Retinal Barrier Breakdown in Rats

Retinas were analyzed for blood-retinal barrier (BRB) breakdown 5 days after intravitreal injection of sCD40. The BRB breakdown was assessed by intravenous injection of fluorescein isothiocyanate (FITC)-conjugated dextran and tracking of the dye in the retinas, as previously described [3]. BRB breakdown was calculated using the following equation, with the results being expressed in µL/g/h.

$$\frac{Retinal\;FITC-Dextran\;\left(µg\right)\;÷\;retinal\;weight\;(g)}{Plasma\;FITC-Dextran\;concentration\;\left(µg/µL\right)\times circulation\;time\;}$$

### 4.5. Human retinal Müller Glial Cell and Human Retinal Microvascular Endothelial Cell Cultures

Human retinal Müller glial cells (MIO-M1) (a generous gift from Prof. A. Limb, Institute of Ophthalmology, University College, London, UK) and retinal microvascular endothelial cells (HRMECs) (Cell Systems Corporation, Kirkland, WA, USA) were obtained and cultured as previously described [3,4,5,6]. In the cell experiments the following stimuli were used: 100 ng/mL [22,30,31,32] recombinant human sCD40L (Cat No. 6420-CL, R&D Systems), 50 ng/mL recombinant human tumor necrosis factor-α (TNF-α) (Cat No 210-TA, R&D Systems), 50 ng/mL recombinant human vascular endothelial growth factor (VEGF) (Cat No 293-VE-050, R&D Systems), 300 μM of the hypoxia mimetic agent cobalt chloride (CoCl2) (Cat No A1425-L, Avonchem Limited, Nacclesfield, Cheshire, UK), 25 mM glucose (Cat No GL0125100, Scharlau S.L, Gato Prez, Barcelona, Spain). For high-glucose treatment, 25 mM mannitol (Cat No MA01490500, Scharlau S.L, Gato Prez, Spain) was used as a control.

Treatment was performed in the absence or presence of 1 h pretreatment with 10 µM of the nuclear factor-kappa B (NF-κB) inhibitor BAY11-7085 ((E)-3-(4-tert-butylphenylsulfonyl)acrylonitrile (Cat No sc-202490, Santa Cruz Biotechnology Inc., Santa Cruz, CA, USA), 25 μM of the furin-inhibitor dec-CMK (Cat No 3501/1, R&D Systems), 10 μM of the MMP inhibitor ONO-4817 (Cat No sc-203139, Santa Cruz Biotechnology Inc.) or 100 ng/mL of tissue inhibitor metalloproteinase-3 (TIMP-3) (Cat No 973-TM-010, R&D Systems).

After 24 h, cell supernatants were collected and processed for ELISA analysis. Harvested cells were lysed in a radioimmunoprecipitation assay (RIPA) lysis buffer (sc-24948, Santa Cruz Biotechnology, Inc.) for Western blot analysis.

### 4.6. Enzyme-Linked Immunosorbent Assays of Human Vitreous Fluid and Culture Medium

Enzyme-linked immunosorbent assay (ELISA) kits for human VEGF (Cat No DY293B), human monocyte chemotactic protein (MCP)-1/CCL2 (Cat No DY279), human CD40 (Cat No DY632), human matrix metalloproteinaise-9 (MMP-9) (Cat No DY911) and human sICAM-1 (Cat No SCD540) were purchased from R&D Systems. ELISA kit for human high-mobility group box-1 (HMGB1) (Cat No ST51011) was purchased from IBL International GMBH (Hamburg, Germany). The levels of human VEGF, sCD40, and sICAM in vitreous fluid and VEGF, MCP-1, sCD40, MMP-9 and HMGB1 in culture medium were determined using the aforementioned ELISA kits according to the manufacturer’s instructions. The minimum detection limits for VEGF, MCP-1, sCD40, MMP-9, sICAM, and HMGB1 ELISA kits were approximately 12 pg/mL, 9 pg/mL, 12 pg/mL, 10 pg/mL, 96 pg/mL, and 200 pg/mL, respectively.

### 4.7. Western Blot Analysis of Human Vitreous Fluid, Human Retinal Müller Glial Cell and Human Retinal Microvascular Endothelial Cell Lysates and Rat Retinas

Retina and cell lysates were prepared as previously described [3,4,5,6]. Equal amounts of protein were loaded for SDS-PAGE and subsequent transfer to nitrocellulose membranes. To determine the presence of CD40 and sCD40L in the vitreous samples, equal volumes (15 μL) of vitreous fluids were boiled in Laemmli’s sample buffer (1:1, *v*/*v*) under reducing conditions for 10 min before loading.

Immunodetection was performed with the use of a rabbit polyclonal anti-CD40 antibody (1:1000, ab13545, Abcam), rabbit polyclonal anti-CD40L antibody (1:1000, ab65854, Abcam), rabbit polyclonal anti-CD40L antibody (1:1000, ab2391, Abcam), rabbit polyclonal anti-p65 subunit of nuclear factor-kappa B (phospho NF-κB) antibody (1:1000, ab86299, Abcam), rabbit monoclonal anti-phospho-extracellular signal-regulated kinase (ERK)1/2 antibody (1:1000, MAB1018, R&D Systems), mouse monoclonal anti-VEGF antibody (1:750, MAB293, R&D Systems), mouse monoclonal anti-intercellular adhesion molecule-1 (ICAM-1) antibody (1:100, sc-8439, Santa Cruz Biotechnology Inc.), mouse monoclonal anti-vascular cell adhesion molecule-1 (VCAM-1) antibody (1:100, sc-13160, Santa Cruz Biotechnology Inc.), mouse monoclonal anti-TNF-α antibody (1:1000, MAB610, R&D Systems), mouse monoclonal anti-glial fibrillary acidic protein (GFAP) antibody (1:1000, ab4648, Abcam) and rabbit polyclonal anti-caspase-3 antibody (1:1000, sc-7148, Santa Cruz Biotechnology Inc.).

Nonspecific binding sites on the nitrocellulose membranes were blocked (1.5 h, room temperature) with 5% non-fat milk made in Tris-buffered saline containing 0.1% Tween-20 (TBS-T). Three TBS-T washings (5 min each) were performed before the secondary antibody treatment at room temperature for 1 h. The secondary antibodies included goat anti-rabbit immunoglobulin (SC-2004) and goat anti-mouse immunoglobulin (SC-2005) (1:2000, Santa Cruz Biotechnology Inc.). To verify equal loading, membranes were stripped and reprobed with β-actin-specific antibody (1:3000, sc-47778, Santa Cruz Biotechnology Inc.).

### 4.8. Cell Adhesion Assay

To determine leukocyte adhesion to stimulated HRMEC monolayers, we used the CytoSelect Leukocyte-endothelium adhesion kit (Cat No CBA-210, Cell Biolabs Inc., San Diego, CA, USA). The assay protocol was followed as described previously [4]. Briefly, 2 × 10^5^ HRMECs were seeded into 0.2% gelatin-coated 24-well plates. After reaching a confluent monolayer, overnight starved endothelial cells were treated either with 25 ng/mL recombinant human TNF-α as a positive control, 100–300 ng/mL recombinant human sCD40L or the potent CD40-CD40L inhibitor DRI-C21045 (50 μM) (MedChemExpress LLC, Monmouth Junction, NJ, USA) for 24 h or 4 h pretreatment with DRI-C21045 followed by TNF-α treatment. Next, 5 × 10^5^ fluorescent-LeukoTracker labelled monocytic THP-1 cells (American Type Culture Collection, Manassas, VA, USA) were added on top of the treated HRMECs monolayer for 30 min. After washing, the remaining adherent THP-1 cells were lysed and fluorescence was measured using a spectraMax Gemini-XPS (Molecular Devices, San Jose, CA, USA) with excitation and emission wavelengths of 485 nm and 538 nm, respectively.

### 4.9. Flow Cytometric Detection of CD40 Expression on HRMECs

HRMECs were detached using the Lonza trypsin/EDTA kit specifically developed for endothelial cells and allowed to recover from trypsin treatment for 45 min at room temperature. Afterwards, cells were incubated in PBS with human FcR block (Miltenyi Biotec, Bergisch Gladbach, Germany) to prevent nonspecific staining and 1 µL Zombie Aqua 510 (Biolegend; San Diego, CA, USA) for selection of live cells (1 × 10^5^ cells; 100 µL/tube). After a wash step with 1 mL flow cytometry buffer (PBS + 2% [*v*/*v*] FCS + 2 mM [*w*/*v*] EDTA), in-house titrated mouse anti-human CD40/TNFRSF5 antibody (R&D systems, Minneapolis, MN; Cat No. MAB6321; clone 82111) was added at a concentration of 2 µg/mL. Staining was finalized by incubation with secondary antibody (10 µg/mL Alexa Fluor 488-labeled goat anti-mouse IgG cross-absorbed; cat A-11001 Invitrogen, Carlsbad, CA) for 30 min on ice in the dark. Fixed cell suspensions were analyzed with a BD LSRFortessa X-20 (BD Biosciences, San Jose, CA, USA). Data analysis was performed using FlowJo Software version 10.7.1.

### 4.10. Endothelial Cell Proliferation Assay

The ability of sCD40L to stimulate proliferation of HRMECs was examined as previously described [44]. HRMECs were seeded at 5 × 10^4^ cells/mL (100 µL/well) in EBM-2 medium supplemented with 1% (*v*/*v*) FCS (proliferation medium) in a clear flat bottom 96-well plate and incubated overnight at 37 °C and 5% CO_2_. The next day, cells were starved in EBM-2 basal medium for 4 h at 37 °C and 5% CO_2_. Afterwards, HRMECs were stimulated with proliferation medium only, or medium supplemented with 10 ng/mL human recombinant VEGF165 (R&D systems) or human recombinant sCD40L (R&D systems) at concentrations of 125 ng/mL, 31.3 ng/mL, or 7.8 ng/mL. After 3 days, a luminescence ATP detection assay was performed according to the manufacturer’s instructions (Perkin Elmer, Waltham, MA). Basal proliferation was subtracted from the individual luminescence values of the stimulated conditions to determine the net increase in proliferation.

### 4.11. Endothelial Cell Spheroid Sprouting Assay

The in vitro angiogenic effect of sCD40L was explored in a spheroid sprouting assay. Spheroids were created through the hanging droplet method as previously described [45]. Briefly, drops containing 1 × 10^3^ HRMECs (25 µL) from a mixture of 20% (*v*/*v*) methylcellulose (Sigma-Aldrich, St. Louis, MO, USA; in EBM-2 basal medium) and HRMECs (4 × 10^4^ cells/mL) resuspended in EBM-2 cell culture medium were plated on a Petri dish and subverted. After 24 h at 37 °C and 5% CO_2_, single spheroids were collected. After centrifugation, spheroids were resuspended in a mixture of 5.5 mg/mL methylcellulose in basal EBM-2 medium, 2.5 mg/mL NaHCO_3_, 1 mg/mL collagen type I (BD Biosciences) and 10 mM NaOH and transferred to a clear flat bottom 96-well plate (100 µL/well) and left at 37 °C, 5% CO_2_ for 30 min to allow collagen to solidify. Thereafter, spheroids were incubated for 16 h at 37 °C and 5% CO_2_ with buffer (EBM-2 + 3% [*v*/*v*] FCS) or buffer supplemented with 10 ng/mL human recombinant VEGF165 or human recombinant sCD40L at concentrations of 500 ng/mL, 125 ng/mL, 31.3 ng/mL, or 7.8 ng/mL. Imaging of the spheroids was performed through a 10× objective on an inverted Axiovert 200M microscope (Carl Zeiss Microscopy GmbH, Oberkochen, Germany). The average number of sprouts per spheroid and the average cumulative sprout length per spheroid were determined with the use of Fiji (ImageJ) software version 1.52v (NIH, Bethesda, MD, USA).

### 4.12. Müller Glial Cell Proliferation Assay

Human retinal Müller glial cells were seeded in a 12-well plate at 5 × 10^4^ cells/well and serum-starved overnight. Cells were then stimulated with 10 ng/mL VEGF (Cat No 293-VE-050, R&D Systems), or 100 ng/mL human recombinant sCD40L (Cat No. 6420-CL, R&D Systems) in starved medium. After 48 h, cells were harvested, and live cells (trypan blue negative cells) were counted with phase-contrast microscopy. Proliferation was expressed relative to the cell numbers counted in the control wells.

### 4.13. Statistical Analysis

Data were collected, stored and managed in a spreadsheet using Microsoft Excel 2010^®^ software. Data were analyzed and figures prepared using SPSS^®^ version 21.0 (IBM Inc., Chicago, IL, USA) and GraphPad Prism software 9.3.0. Tests for normality were done using the Shapiro–Wilk test and Q-Q plots. The normally distributed data were reported as mean ± standard deviation (SD) and range and illustrated using bar charts showing the standard deviations, whereas not normally distributed data were presented as median (interquartile range) (IQR) with box and whisker plots figures. Consequently, one-way ANOVA and independent *t*-test or Kruskal–Wallis and Mann–Whitney tests (applying Bonferroni correction where necessary) were used to test the differences between the groups for normally distributed data and non-normally distributed data, respectively. Furthermore, Pearson’s and Spearman’s correlation analyses were performed for normally and not normally distributed data, respectively. Any output with a *p*-value below 0.05 was interpreted as an indicator of statistical significance.

## 5. Conclusions

Our results suggest that the CD40L-CD40 signaling pathway is an upstream regulator of inflammatory and angiogenic responses involved in the development of DR and might be involved in the maintenance of inflammation-driven angiogenesis in PDR. The CD40L-CD40 pathway could also have a biomarker potential. In addition, these findings improve our understanding of the molecular links between inflammation and angiogenesis in PDR. On the basis of experiments in rats, the modulation of the CD40L-CD40 pathway may become a novel target for therapeutic intervention to treat inflammation-driven breakdown of the BRB and angiogenesis in patients with DR. However, further investigations and alternative techniques are needed to explore the signaling pathways that provide the mechanistic link between inflammation and angiogenesis induced by the interaction between CD40L and its counter-receptor CD40 in the diabetic retina.

## Figures and Tables

**Table 1:** Comparisons of CD40, vascular endothelial growth factor (VEGF) and soluble intercellular adhesion molecule-1 (sICAM-1) levels in vitreous samples from patients with proliferative diabetic retinopathy (PDR) and nondiabetic patients with rhegmatogenous retinal detachment (RD).

	PDR (*n* = 38) Median (IQR)	RD (*n* = 32) Median (IQR)	*p*-Value (Mann–Whitney Test)
CD40	173.00 (90.4–290.63)	21.65 (<DL–97.38)	<0.001 *
(pg/mL)			
VEGF	446.25 (47.7–1013.02)	<DL (<DL–56.6)	<0.001 *
(pg/mL)			
sICAM-1	11.07 (5.29–17.56)	3.07 (1.67–9.48)	<0.001 *
(ng/mL)			

* Statistically significant at 5% level of significance. DL = detection limit; IQR = interquartile range (Q1–Q3).

## Data Availability

Data are available from the authors upon request.
